# Comparative Analysis of Antibiotic Resistance and Biofilm Characteristics of Two Major *Enterococcus* Species from Poultry Slaughterhouses in South Korea

**DOI:** 10.3390/vetsci11040180

**Published:** 2024-04-16

**Authors:** Yongwoo Son, Yeung Bae Jin, Eun-Jeong Cho, Ae Ra Park, Rochelle A. Flores, Binh T. Nguyen, Seung Yun Lee, Bujinlkham Altanzul, Kwang Il Park, Wongi Min, Woo H. Kim

**Affiliations:** 1 College of Veterinary Medicine, Gyeongsang National University, Jinju 52828, Republic of Korea; ywson@gnu.ac.kr (Y.S.); ybjin@gnu.ac.kr (Y.B.J.); floresrochellea@gmail.com (R.A.F.); thanhbinhcnty@gmail.com (B.T.N.); seungyun0218@gnu.ac.kr (S.Y.L.); bujinlkham_1221@gnu.ac.kr (B.A.); kipark@gnu.ac.kr (K.I.P.); wongimin@gnu.ac.kr (W.M.); 2 Gyeongnam Veterinary Service Laboratory, Jinju 52733, Republic of Korea; vet9714@korea.kr (E.-J.C.); zippy6584@korea.kr (A.R.P.)

**Keywords:** *Enterococcus faecalis*, *Enterococcus faecium*, antibiotic resistance, biofilm formation, virulence genes, multidrug resistance, poultry industry

## Abstract

**Simple Summary:**

This study focused on the antibiotic resistance and biofilm characteristics of two predominant species of *Enterococcus*, *E. faecalis* and *E. faecium*, isolated in poultry slaughterhouses in South Korea. *E. faecium* showed a broader range of antibiotic resistance, particularly to linezolid and rifampicin. A high level of multidrug resistance was also observed in *E. faecalis* (95.8%) and *E. faecium* (93.8%). *E. faecalis* formed more robust biofilms than *E. faecium*. We also identified several specific genes (*cob*, *ccf*, and *sprE*) that were associated with the strength of the biofilm. There was no general correlation between antibiotic resistance and biofilm strength found in the isolates used in this study. These findings point to the potential health risks of these drug-resistant bacteria as they can spread from poultry to humans through the food supply chain.

**Abstract:**

The spread of antibiotic-resistant *Enterococcus* in the poultry industry poses significant public health challenges due to multidrug resistance and biofilm formation. We investigated the antibiotic resistance profiles and biofilm characteristics of *E. faecalis* and *E. faecium* isolates from chicken meat in poultry slaughterhouses in South Korea. Ninety-six isolates (forty-eight each of *E. faecalis* and *E. faecium*) were collected between March and September 2022. Both species were analyzed using MALDI-TOF, PCR, antibiotic susceptibility testing, and biofilm assays. A high level of multidrug resistance was observed in *E. faecalis* (95.8%) and *E. faecium* (93.8%), with *E. faecium* exhibiting a broader range of resistance, particularly to linezolid (52.1%) and rifampicin (47.9%). All *E. faecalis* isolates formed biofilm in vitro, showing stronger biofilm formation than *E. faecium* with a significant difference (*p* < 0.001) in biofilm strength. Specific genes (*cob*, *ccf*, and *sprE*) were found to be correlated with biofilm strength. In *E. faecium* isolates, biofilm strength was correlated with resistance to linezolid and rifampicin, while a general correlation between antibiotic resistance and biofilm strength was not established. Through analysis, correlations were noted between antibiotics within the same class, while no general trends were evident in other analyzed factors. This study highlights the public health risks posed by multidrug-resistant enterococci collected from poultry slaughterhouses, emphasizing the complexity of the biofilm-resistance relationship and the need for enhanced control measures.

## 1. Introduction

Biofilm-producing antibiotic-resistant bacteria in food pose both direct and indirect risks to consumers. The direct risk from these potential bacteria contributes to foodborne infections that occur through inadequate cooking or cross-contamination through improper handling and are always considered challenging. On the other hand, horizontal gene transfer leading to increased resistance in the bacterial population is a major consequence of indirect risk.

Enterococci are opportunistic pathogens that colonize the intestinal tract of humans and animals, including poultry [1]. They have the potential to contaminate food, acting as a vector for the transfer of drug-resistant genes from farms to dining tables to clinics [2]. Enterococci exhibit a remarkable ability to adapt to harsh environments: they can withstand extreme temperatures, ranging from 10 °C to over 45 °C, and tolerate high salt concentrations and pH levels [3]. These characteristics, along with their capacity for biofilm formation, reduce the disinfection efficiency of surfaces that these bacteria colonize, thereby elevating the risk of dissemination [4].

Antibiotic resistance, particularly biofilm-mediated resistance in enterococci, presents a significant concern. A rising challenge is multidrug-resistant (MDR) enterococci tied to nosocomial infections. Enterococci inherently resist many antibiotics, including cephalosporins, aminoglycosides, trimethoprim-sulfamethoxazole, lincosamides, and quinolones. For instance, this intrinsic resistance to quinupristin/dalfopristin in *E. faecalis* is attributed to the *lsa* gene [5,6]. Furthermore, enterococci rapidly acquire resistance to antibiotics through mutations (various levels of resistance to aminoglycosides) or genetic transfers through plasmids, transposons, or integrons (resistance to glycopeptides, mainly vancomycin) [7]. Comparatively, *E. faecium* exhibits a higher antibiotic resistance than *E. faecalis*. For instance, the inherent resistance of enterococci to aminoglycosides is due to their cell wall’s low permeability to these large molecules [8]. It is more prevalent in *E. faecium* than *E. faecalis* (49.2% and 8.9%, respectively) [9]. The innate resistance to β-lactam results from overexpressed penicillin-binding proteins with low affinity for β-lactams [10]. Consequently, *E. faecalis* is 10–100 times more resistant to penicillin than streptococci, indicating a higher minimum inhibitory concentration (MIC). Moreover, *E. faecium* tends to be 4–16 times more resistant than *E. faecalis* [11]. Especially, vancomycin-resistant enterococci (VREs) continue to gain resistance to various antibiotics, underscoring the pressing need for new antibiotics [12].

Antibiotic-resistant enterococci (ARE) can be transmitted to the human gut through the consumption of contaminated foods, particularly of animal origin, and the environment, including people and animals. Given that enterococci are not traditionally classified as foodborne pathogens, establishing zoonotic links via the food chain is complex. Nonetheless, the potential for food-associated AREs to facilitate the transmission of antibiotic resistance into healthcare settings remains a significant concern [13]. Studies have pointed to food chains, especially beef, pork, and poultry meat, as potential reservoirs of AREs [14,15,16]. While cooking significantly reduces the levels of enterococci in meat, cross-contamination of edible carcass tissues during slaughter poses a notable food safety risk [13,17]. In the study of foodborne AREs, poultry meat might pose a more significant threat than beef or pork due to the higher prevalence of resistance genes in AREs isolated from retail chickens [18]. 

The ability of enterococci to form biofilms increases their resilience, adding to persistent infections and contamination in the environment and food industry [12]. Bacterial biofilm formation is an integral part of many diseases, both in humans and animals, with >60% of bacterial infections estimated to be associated with biofilm [4]. Forming a biofilm enhances bacterial viability, increases antibiotic resistance, and provides stability against diverse environments, including the host’s immune response [19]. Hence, ongoing studies are exploring genes associated with biofilm formation. Biofilm development follows four stages: attachment, microcolony formation, maturation (influenced by quorum sensing), and dispersal. Virulence factors play a substantial role in enterococcal pathogenesis through biofilm production. Biofilm-related virulence factors can be categorized into two groups: secreted factors, such as cytolysin, secreted antigen A, and gelatinase; and cell surface factors, which encompass pili, microbial surface components recognizing adhesive matrix molecules (MSCRAMMs), and aggregation substances (ASs) [20]. Several genes associated with enterococcal biofilm formation have been identified, including *agg* [21] (involved in surface attachment), *efaA* [22] (cell adhesion), *srt* [23] (microcolony formation), *bop* [24], *cob* [25], *ccf* [26] (quorum-sensing molecules), and *gelE/sprE* [27] (biofilm growth and maturation). 

The ability of enterococci to resist multiple drugs, in conjunction with their potent biofilm-forming capabilities, poses a significant challenge to public health. However, there is limited research on ARE isolates and their biofilm formation in South Korea. This study examined the correlation between antibiotic resistance profiles, biofilm formation, and virulence genes associated with this process in *E. faecalis* and *E. faecium* isolated from chicken meat in poultry slaughterhouses in 2022.

## 2. Materials and Methods

### 2.1. Bacterial Isolates and Culture Media

From March to September 2022, *Enterococcus* isolates were collected regularly every two weeks from chicken meat at three poultry slaughterhouses in Gyeongsangnam-do until 48 isolates each of *E. faecalis* and *E. faecium* were obtained. Swab samples were taken from randomly selected parts of the carcasses after evisceration. Isolation was performed by mixing with Nasco swab solution (1 mL) and azide dextrose broth (9 mL) (BD Difco, Franklin Lakes, NJ, USA). Following incubation at 37 °C for 16–20 h, an inoculum of broth culture was cultivated in m-Enterococcus agar (mEA) (BD Difco, USA) plates with incubation for 48 h at 37 °C. Isolated pink colonies were cultivated in mEA plates under the same incubation conditions. Isolated single colonies that were morphologically suggestive of enterococci were subsequently grown in tryptic soy agar (TSA) (BD Difco, USA) plates at 37 °C for 48 h. They were finally confirmed as *Enterococcus* spp. by a MALDI Biotyper system (Bruker Corporation, Billerica, MA, USA) and multiplex PCR.

### 2.2. Antibiotic Susceptibility Test

A disc diffusion test was conducted to assess the antibiotic resistance patterns of *Enterococcus* isolates. The bacterial concentration was adjusted to 0.5 McFarland standard using a DEN-1B densitometer (Biosan, Riga, Latvia) and then spread on Muller–Hinton agar (BD Difco, USA). The antibiotics employed for the sensitivity test included: ampicillin (AMP: 30 μg), ciprofloxacin (CIP: 5 μg), doxycycline (DOX: 30 μg), erythromycin (ERY: 15 μg), linezolid (LZD: 30 μg), minocycline (MIN: 30 μg), penicillin (PEN: 10 U), quinupristin/dalfopristin (Q/D: 15 μg), rifampicin (RIF: 5 μg), and vancomycin (VAN: 30 μg) (Table 1). MIC breakpoints for each antibiotic were determined in accordance with CLSI VET01S guidelines [28]. MDR was defined as acquired non-susceptibility to at least one agent in three or more antibiotic categories, as previously outlined by [29].

### 2.3. Microtiter Plate Biofilm Assay

Biofilm formation was examined using the method described by Christensen with few modifications [30]. The confirmed isolates of *Enterococcus* spp. were inoculated onto TSA plates and incubated at 37 °C for 24 h to attain a single colony. A uniform cell suspension was produced from a single confirmed enterococcal colony on a TSA plate with sterile physiological saline, and the opacity was adjusted to a 0.5 McFarland standard. A 20 μL aliquot of the vortexed bacterial suspension was added to 180 μL of tryptic soy broth (TSB) containing 0.5% glucose in a 96-well polystyrene microtiter plate and incubated again at 37 °C for 24 h. Following incubation, the excessive broth was gently discarded, and each well was washed three times with 300 μL of sterile physiological saline. The biofilm was fixed for 20 min using 150 μL of methanol, and the microtiter plate was emptied, inverted, and dried for roughly 30 min. An amount of 150 μL of 2% crystal violet was added, left at room temperature for 15 min, and then rinsed with tap water. To solubilize the dye, 150 μL of 33% acetic acid was added to each well and left to react for 30 min at room temperature. The optical density (OD) was subsequently measured at 540 nm using a Multiskan SkyHigh Microplate Spectrophotometer (Thermo Fisher Scientific, Waltham, MA, USA). The cut-off value (ODc) was calculated according to Stepanović [31] to classify isolates by their ability to form biofilms. It was categorized as no biofilm producer (OD_540_ ≤ ODc), weak biofilm producer (ODc < OD_540_ ≤ 2×ODc), moderate biofilm producer (2×ODc < OD_540_ ≤ 4×ODc), and strong biofilm producer (4×ODc < OD_540_).

### 2.4. Polymerase Chain Reaction

DNA from the isolates was extracted using the Exgene™ Cell SV kit (GeneAll Biotechnology, Seoul, Republic of Korea) following the manufacturer’s instructions. PCR amplification was performed on the extracted DNA using a T-100 thermal cycler (Bio-Rad Laboratories, Hercules, CA, USA) to confirm the species and analyze the biofilm-associated gene characteristics in the isolates. Species-specific identification of *Enterococcus* spp. was achieved through multiplex PCR, simultaneously amplifying the 16S rRNA gene and utilizing species-specific primer sets to enable precise differentiation among *Enterococcus* spp. The specific primer sequences and corresponding annealing conditions for the PCR are presented in Table 2. The amplification conditions were as follows: initial denaturation step at 94 °C for 10 min, followed by 35 cycles of denaturation at 94 °C for 1 min, annealing for 30 s, and extension at 72 °C for 1 min, concluding with a final extension of 5 min at 72 °C. The PCR products were subjected to electrophoresis in a 2% agarose gel with TAE buffer using a Mupid One system (Takara Bio, Kusatsu, Japan). The bands were visualized and documented using a GelDoc system (Bio-Rad Laboratories, USA).

### 2.5. Statistical Analysis

Data analysis involved the use of the Mann–Whitney test, Fisher’s exact test, and Spearman’s rank correlation analysis. Statistical analyses were performed using GraphPad Prism version 9.5.0 (GraphPad Software, San Diego, CA, USA) and R version 4.3.1 (R Foundation for Statistical Computing, Vienna, Austria). A *p*-value of <0.05 was considered statistically significant (* < 0.05, ** < 0.01, *** < 0.001).

## 3. Results

### 3.1. Identification of Enterococcus *spp.*

In the study, the MALDI-TOF analysis has shown that 96 isolates were *Enterococcus* spp. The PCR for *enterococcal* 16S rRNA and species-specific primers were subsequently conducted using total DNA from isolates, showing accurate amplification at 733 bp across all 96 isolates. Additionally, for the specific species-detection of *E. faecalis* and *E. faecium*, 360 bp and 214 bp were correctly amplified in all 48 isolates of *E. faecalis* and *E. faecium*, respectively.

### 3.2. Antibiotic Susceptibility Profile

**Figure 1:**
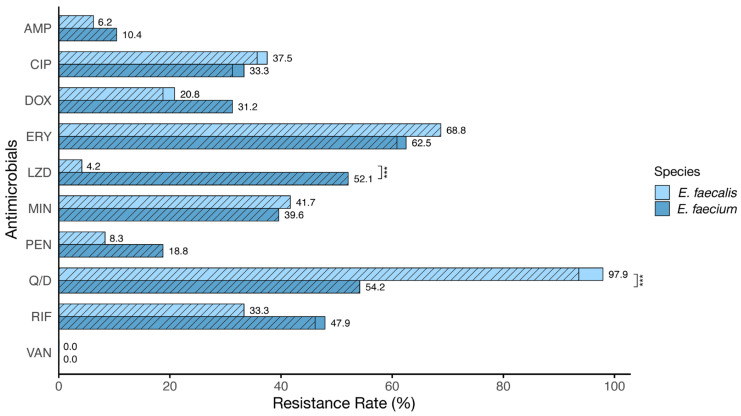
Distribution of antibiotic-resistant *E. faecium* and *E. faecalis* isolates used in this study. The hatched sections represent the percentage of multidrug-resistant isolates within the resistant categories. The antibiotics tested include ampicillin (AMP), ciprofloxacin (CIP), doxycycline (DOX), erythromycin (ERY), linezolid (LZD), minocycline (MIN), penicillin (PEN), quinupristin/dalfopristin (Q/D), rifampicin (RIF), and vancomycin (VAN). *** indicates a significant difference in antibiotic resistance between *E. faecalis* and *E. faecium* (*p* < 0.001; Fisher’s exact test).

Figure 1 illustrates the antibiotic resistance profile analysis in *E. faecalis* and *E. faecium* isolates. For *E. faecalis*, resistance rates were observed as follows: Q/D (97.9%), ERY (68.8%), MIN (41.7%), CIP (37.5%), RIF (33.3%), DOX (20.8%), PEN (8.3%), AMP (6.2%), LZD (4.2%), and VAN (0.0%). For *E. faecium*, the resistance rates were ERY (62.5%), Q/D (54.2%), LZD (52.1%), RIF (47.9%), MIN (39.6%), CIP (33.3%), DOX (31.2%), PEN (18.8%), AMP (10.4%), and VAN (0.0%). *E. faecalis* showed higher resistance to CIP, ERY, MIN, and Q/D, while *E. faecium* showed higher resistance to AMP, DOX, LZD, PEN, and RIF. Resistance to Q/D and LZD significantly differed between species (*p* < 0.001).

Antibiotic resistance profiles of *E. faecalis* and *E. faecium* can be found in Supplementary Table S1. For *E. faecalis*, resistance to a single antibiotic was found in 5 isolates (10.4%), while 43 isolates (89.6%) were resistant to more than two antibiotics. The most common resistance pattern observed was CIP-ERY-MIN-Q/D in nine isolates (18.6%). For *E. faecium*, one isolate was pan-susceptible, four isolates (8.3%) showed resistance to a single antibiotic, and forty-three isolates (89.6%) were resistant to more than two antibiotics. The most common resistance pattern observed was ERY-LZD-Q/D-RIF in seven isolates (14.6%). Upon analyzing multidrug resistance, *E. faecalis* exhibited an average multiple antibiotic resistance (MAR) index of 0.32, with 46 out of 48 isolates (95.8%) displaying an MDR pattern. Similarly, *E. faecium* demonstrated an average MAR index of 0.35, with 45 out of 48 isolates (93.8%) exhibiting an MDR pattern (Supplementary Tables S2 and S3). There was no significant difference in the MAR index between *E. faecalis* and *E. faecium*.

### 3.3. Biofilm Formation

**Figure 2:**
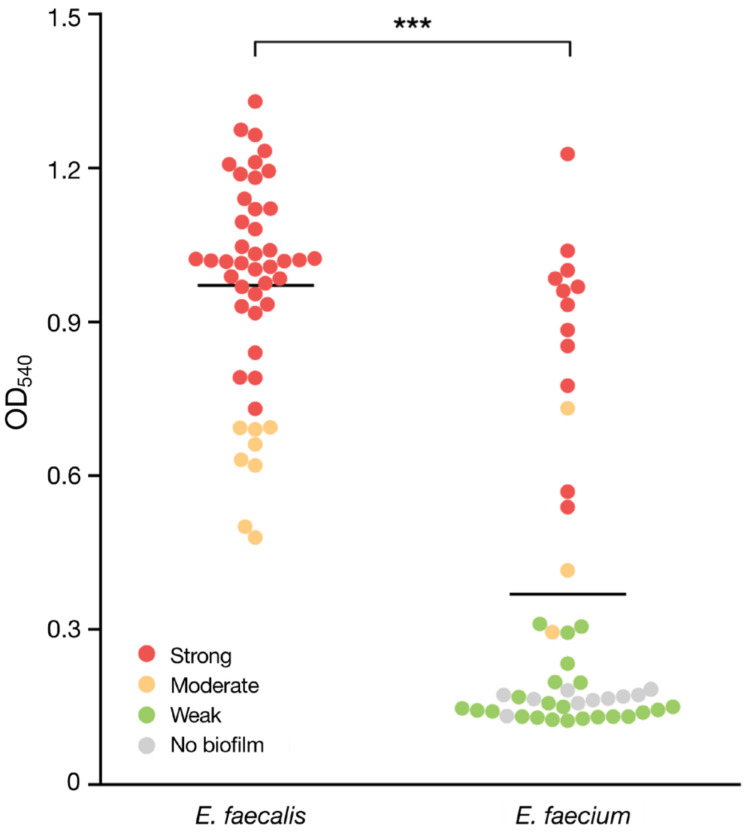
Comparison of OD_540_ values representing biofilm strength in enterococcal isolates. The OD cut-off was calculated individually for each microtiter plate. For *E. faecalis*, two isolates (OD_540_ = 1.65, 3.35) with higher values than others were excluded from the graph. The solid line indicates the average OD_540_ for each species. *** indicates a significant difference in biofilm strength between *E. faecalis* and *E. faecium* (*p* < 0.001, Mann–Whitney test).

In the analysis of biofilm formation abilities in *E. faecalis* and *E. faecium*, 40 out of the 48 isolates (83.3%) of *E. faecalis* displayed strong biofilm formation, while the remaining 8 isolates (16.7%) were moderate. For *E. faecium*, 12 isolates (25%) exhibited strong biofilm formation, 3 isolates (6.3%) showed moderate biofilm formation, 23 isolates (47.9%) showed weak biofilm formation, and the biofilm was not detected using a microtiter-based assay for 10 isolates (20.8%) (Figure 2). The mean OD_540_ value was 1.03 for *E. faecalis* and 0.37 for *E. faecium*. A significant difference between the two species (*p* < 0.001) was observed.

### 3.4. Screening Genes Involved in Biofilm Formation

To examine genes associated with biofilm formation, 98 *Enterococcus* isolates were screened by PCR for the seven genes (Table 3 and Table 4). The prevalence rates of genes of *E. faecalis* are observed as follows: *agg* (77.1%), *efaA* (0.0%), *bop* (100%), *srt* (95.8%), *sprE* (100%), *cob* (100%), and *ccf* (100%). Genes of *E. faecium* are observed as follows: *agg* (79.2%), *efaA* (0.0%), *bop* (85.4%), *srt* (91.7%), *sprE* (56.3%), *cob* (33.3%), and *ccf* (54.2%). Fisher’s exact test was employed to evaluate the association between each gene and biofilm formation. There was a significant positive correlation (*p* < 0.05) between *sprE* and biofilm strength, specifically in *E. faecium* isolates. Despite no statistical significance detected between the two species, all tested *E. faecalis* isolates were positive for *cob*, which is a particularly strong biofilm former. In contrast, approximately two-thirds of *E. faecium* strains showed negativity for this gene. 

### 3.5. Antibiotic Resistance across Biofilm Strength

**Figure 3:**
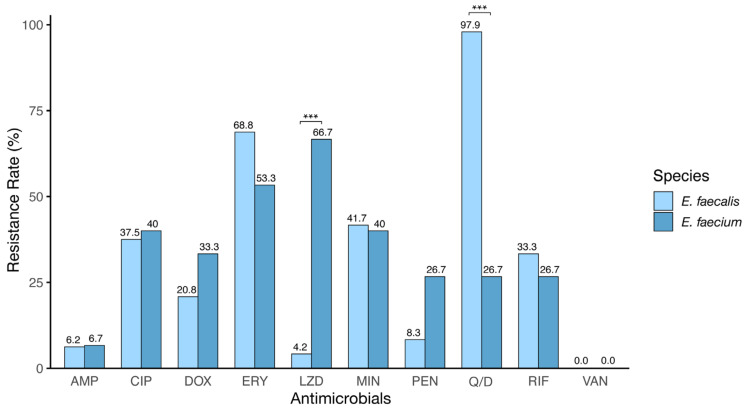
Distribution of antibiotic-resistant *E. faecium* and *E. faecalis* isolates forming a moderate and strong biofilm. The antibiotics tested include ampicillin (AMP), ciprofloxacin (CIP), doxycycline (DOX), erythromycin (ERY), linezolid (LZD), minocycline (MIN), penicillin (PEN), quinupristin/dalfopristin (Q/D), rifampicin (RIF), and vancomycin (VAN). *** indicates a significant difference in antibiotic resistance between *E. faecalis* and *E. faecium* (*p* < 0.001, Fisher’s exact test).

Figure 3 presents the resistance profiles of isolates exhibiting moderate and strong biofilm strengths to observe the correlation between biofilm strength and antibiotic resistance. In *E. faecalis*, the resistance was most prevalent to Q/D, followed by ERY, MIN, and CIP, with lower levels of resistance to AMP, PEN, and LZD and no resistance detected to VAN. For *E. faecium*, the highest resistance was against LZD, then DOX, CIP, MIN, RIF, and PEN, with lower resistance to Q/D and AMP, and similarly, no resistance to VAN was observed. Notably, *E. faecium* isolates forming moderate to strong biofilms exhibited higher resistance to AMP, LZD, and PEN than other isolates. Inter-species analysis revealed significant differences in LZD and Q/D resistance (*p* < 0.001). Furthermore, *E. faecalis* demonstrated elevated resistance to ERY, MIN, Q/D, and RIF, while *E. faecium* displayed higher resistance to AMP, CIP, DOX, LZD, and PEN.

### 3.6. Correlations between Studied Factors

**Figure 4:**
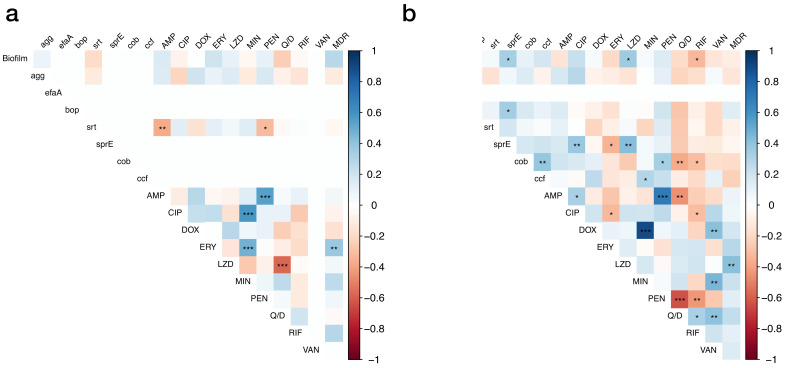
A correlation plot of enterococcal biofilm formation, genes involved in biofilm formation, and antibiotic resistance used in this study. (**a**) Correlation plot of *E. faecalis.* (**b**) Correlation plot of *E. faecium*. The correlation coefficient was calculated by Spearman’s correlation test. The color of each cell indicates the correlation coefficient, with a positive correlation representing blue and a negative correlation representing red. Statistical significance is denoted by asterisks: * *p* < 0.05, ** *p* < 0.01, *** *p* < 0.001.

We analyzed the correlations between phenotypes and genotypes analyzed in the experiment (Figure 4). As a result of the analysis, several correlations were observed for *E. faecalis*, including positive correlations between ERY and MDR. Several correlations were also observed for *E. faecium*, including a positive correlation between biofilm strength and LZD resistance as well as between LZD and MDR. Additionally, there was a negative correlation between biofilm strength and RIF resistance.

## 4. Discussion

Understanding the prevalence of AREs in food animals and the food supply chain is essential for assessing the potential threat to human health. This research analyzed the antibiotic resistance patterns and biofilm characteristics in the two predominant species of *Enterococcus* found in South Korean poultry, providing insights into their implications for public health. 

The antibiotic susceptibility test results showed distinct antibiotic resistance patterns between *E. faecium* and *E. faecalis. Enterococcus faecium* demonstrated slightly higher resistance to cell wall synthesis inhibitors, such as ampicillin and penicillin, than *E. faecalis*. While no isolates were resistant to vancomycin, 23% of *E. faecium* isolates exhibited intermediate resistance to vancomycin, suggesting a potential emergence in VREs. For nucleic acid inhibitors, *E. faecalis* showed slightly higher resistance to ciprofloxacin, and *E. faecium* showed slightly higher resistance to rifampicin. Resistance rates to protein synthesis inhibitors erythromycin and minocycline were similar in both species, while *E. faecium* showed slightly higher resistance to doxycycline. 

A significant correlation in resistance patterns was observed within the same class of antibiotics. For example, a relationship was noted in the resistance to erythromycin and minocycline. However, when comparing *E. faecium* and *E. faecalis*, the distribution of these resistance patterns was notably distinct, particularly in linezolid and quinupristin/dalfopristin—key antibiotics in managing VREs. *E. faecalis* shows a negative correlation with these antibiotics (*p* < 0.001), differing from that of *E. faecium*. This resistance pattern of *E. faecalis* is likely due to the *lsa* gene, which confers innate resistance to quinupristin/dalfopristin [5]. 

The trends of antibiotic resistance in our study are similar to those found in domestic enterococci from 2010 to 2019 [33]. However, excluding linezolid, the absolute numbers were lower compared with research conducted in China in 2022 [34] and Zambia in 2023 [35]. Comparatively, resistance to ampicillin, erythromycin, linezolid, and quinupristin/dalfopristin in enterococcal isolates was higher than those reported in national antibiotic resistance surveillance [33]. *E. faecium* exhibited a significantly increased resistance rate to linezolid compared with previous studies [15,36]. This indicates that these resistant isolates are prevalent in poultry slaughterhouses in South Korea. Enterococci easily acquire and transfer antibiotic-resistant genes, especially those on mobile genetic elements [37]. Thus, the dissemination of resistance from *E. faecium* to *E. faecalis* and other pathogenic bacteria poses a significant risk to public health. 

Further analysis showed *E. faecalis* isolates displayed resistance to an average of 3.19 different antibiotics, with 95.8% being classified as MDR enterococci. Similarly, *E. faecium* isolates exhibited resistance to an average of 3.5 different antibiotics, with 93.8% being identified as MDR enterococci. These rates of MDR are higher than those reported in earlier studies [33,38,39]. The most common resistance patterns, CIP-ERY-MIN-Q/D for *E. faecalis* and ERY-LZD-Q/D-RIF for *E. faecium*, raise concerns as ciprofloxacin and linezolid are prohibited in South Korean poultry [33]. 

Enterococci exchange resistance more easily when forming biofilms, which can lead to problems when biofilms develop on food surfaces [15]. In our study, *E. faecalis* isolates (100%) exhibited a higher ability to form biofilms compared with *E. faecium* (79.2%), with their biofilm strength also being notably stronger (*p* < 0.001). However, the prevalence of biofilm-forming ability varies worldwide. In Saudi Arabia, 86.6% of *E. faecalis* isolates and 80.0% of *E. faecium* isolates were biofilm producers [40]. In Poland, from wild birds, 66.7% of *E. faecalis* isolates and 22.2% of *E. faecium* isolates formed biofilm [41]. These findings suggest that *E. faecalis* more frequently forms biofilm than *E. faecium.* Biofilm formation could play an essential role in the pathogenesis of enterococcal infection. *E. faecalis* forms denser biofilms, enhancing its antibiotic tolerance and contrasting with *E. faecium*, which possesses a higher intrinsic antibiotic resistance [1,12]. The variability in biofilm formation is underscored by the observations of Di Rosa and colleagues, who reported that 95% of *E. faecalis* isolates are biofilm producers as opposed to 29% for *E. faecium* isolates [42]. Despite this, studies report biofilm formation capabilities at 100% for both species [15,38].

Contrary to previous reports, our study found no correlation between biofilm formation and antibiotic resistance patterns except for an association with linezolid and rifampicin resistance in *E. faecium* isolates. Castaño-Arriba et al. have reported that strong biofilm producers showed a higher average number of resistances, yet the relationship between antibiotic resistance and biofilm formation remains controversial [15]. While it is confirmed that biofilm-forming enterococci exhibit higher antibiotic resistance than their planktonic state [1], the relationship between biofilm strength and antibiotic resistance genes is still unclear. 

The roles of several virulence proteins in enterococci and their contribution to biofilm development have been studied [12,20,22]. In this study, there were significant relationships between biofilm strength and the presence of the *cob* gene (*p* < 0.001) and the *ccf* gene (*p* < 0.05). Additionally, in the case of *E. faecium*, a significant positive correlation was observed between biofilm strength and the presence of the *sprE* gene (*p* < 0.05), like in other studies [12,27]. Pheromones like Ccf and Cob facilitate conjugation results in cells [25,26]. *SprE* is co-transcribed with *gelE*, producing extracellular DNA (eDNA) essential for biofilm formation [27]. Our findings reveal that the *ccf*, *cob*, and *sprE* genes appear less frequently in *E. faecium* at 33.3%, 54.1%, and 56.3%, respectively, when contrasted with *E. faecalis*, showing their potential role in biofilm formation. Some studies reported that *efaA* has a clear cooccurrence of *esp*, *sprE*, *agg*, and *efaA* with biofilm formation [18,43]. Our findings, consistent with reference [41,44], did not detect *efaA* in any isolates, indicating variability in *efaA* prevalence across different niches.

In this study, certain limitations are in the methodology of AMR testing and biofilm assay conditions. Selecting single isolated colonies, a common practice for identifying resistant strains, may not encompass all AMR diversity within species. Additionally, the use of CLSI breakpoints, designed for other animal species and anatomic sites, introduces ambiguity in distinguishing resistant strains as these breakpoints may not be directly applicable to our chicken isolates. As an alternative approach, epidemiological cut-off value (ECOFF) can be used in population-level studies, including non-clinical isolates and commensal bacteria from healthy animals. Additionally, while our biofilm assays were performed at 37 °C, we recognize that this may not correspond to the varied temperatures of food surfaces, especially in colder settings like slaughterhouses. This may influence the observed prevalence of AMR and the applicability of our biofilm results to different environments. Future studies should encompass these considerations to better understand AMR and biofilm development across various environments.

In summary, it was found that *E. faecium* typically displays greater resistance to specific antibiotics than *E. faecalis*, with a substantial number of these isolates being multidrug-resistant. *E. faecalis* tends to form a more robust biofilm. However, there was a lack of correlation between biofilm formation and antibiotic resistance, except for linezolid and rifampicin resistance in *E. faecium*. Apart from the *cob*, *ccf*, and *sprE* genes, no significant correlations were found between other genetic factors and biofilm formation.

## 5. Conclusions

In conclusion, we profiled antibiotic resistance and biofilm characteristics among *E. faecalis* and *E. faecium* isolates from chicken meat. While *E. faecalis* tends to form more robust biofilms, which may enhance antibiotic tolerance, *E. faecium* shows a broader range of antibiotic resistance, particularly to linezolid and rifampicin. The study did not find a general correlation between antibiotic resistance and biofilm formation apart from the noted exceptions, indicating the complexity of the resistance–biofilm relationship. The prevalence of multidrug resistance in these isolates, higher than previously reported elsewhere, underscores the critical need for comprehensive surveillance and controls in food production. Future studies should delve into the genetic mechanisms of antibiotic resistance and biofilm formation, exploring potential strategies to mitigate the spread of antibiotic resistance from poultry farms to the human population. In this way, we can manage the persistent challenge posed by *Enterococcus*.

## Figures and Tables

**Table 1:** Antibiotics used in this study.

Antibiotic Class	Antibiotics	Classification	
		WHO	WOAH
Penicillins	Ampicillin	CIA	VCIA
	Penicillin	HIA	VCIA
Quinolones	Ciprofloxacin	CIA	VCIA
Tetracyclines	Doxycycline	HIA	VCIA
	Minocycline	HIA	-
Macrolides	Erythromycin	CIA	VCIA
Oxazolidinones	Linezolid	CIA	-
Streptogramins	Quinupristin/Dalfopristin	HIA	-
Ansamycins	Rifampicin	CIA	VHIA
Glycopeptides	Vancomycin	CIA	-

Critically important antimicrobial (CIA), highly important antimicrobial (HIA), veterinary critically important antimicrobial (VCIA), and veterinary highly important antimicrobial (VHIA).

**Table 2:** Primers used in this study.

Genes	Primer Sequence (5′-3′)		Annealing (°C)	Product Size (bp)	Reference
*Enterococcus* 16S rRNA	F R	TCAACCGGGGAGGGT ATTACTAGCGATTCCGG	55	733	[32]
*E. faecalis*	F R	ACTTATGTGACTAACTTAACC TAATGGTGAATCTTGGTTTGG	55	214	[32]
*E. faecium*	F R	ACAATAGAAGAATTATTATCTG CGGCTGCTTTTTTGAATTCTTCT	55	360	[32]
*agg*	F R	TCTTGGACACGACCCATGAT AGAAAGAACATCACCACGAGC	58	413	[22]
*bop*	F R	GATCGTCTTCGCCATAGTAGG ATACACAACAGCCCTTGGCT	58	312	[22]
*ccf*	F R	GGGAATTGAGTAGTGAAGAAG AGCCGCTAAAATCGGTAAAAT	5	543	[22]
*cob*	F R	GCTTTGTTTGCTGAATGTTCC GACAACTGATGAGGTGCTAG	55	385	[22]
*efaA*	F R	GACAGACCCTCACGAATATG CCAGTTCATCATGCTGTAGTA	52	706	[22]
*sprE*	F R	CTGAGGACAGAAGACAAGAAG GGTTTTTCTCACCTGGATAG	55	432	[22]
*srt*	F R	GTATCCTTTTGTTAGCGATGC TGTCCTCGAACTAATAACCGA	55	612	[22]

**Table 3:** Biofilm-associated genes of *E. faecalis*.

Biofilm Strength	*agg*		*efaA*		*bop*		*srt*		*sprE*		*cob*		*ccf*	
	+ (%)	− (%)	+ (%)	− (%)	+ (%)	− (%)	+ (%)	− (%)	+ (%)	− (%)	+ (%)	− (%)	+ (%)	− (%)
Moderate	6 (12.5)	2 (4.2)	0	8 (16.7)	8 (16.7)	0	8 (16.7)	0	8 (16.7)	0	8 (16.7)	0	8 (16.7)	0
Strong	31 (64.6)	9 (18.8)	0	40 (83.3)	40 (83.3)	0	38 (79.2)	2 (4.2)	40 (83.3)	0	40 (83.3)	0	40 (83.3)	0
Total	37 (77.1)	11 (22.9)	0	48 (100)	48 (100)	0	46 (95.8)	2 (4.2)	48 (100)	0	48 (100)	0	48 (100)	0

**Table 4:** Biofilm-associated genes of *E. faecium*.

Biofilm Strength	*agg*		*efaA*		*bop*		*srt*		*sprE*		*cob*		*ccf*	
	+ (%)	− (%)	+ (%)	− (%)	+ (%)	− (%)	+ (%)	− (%)	+ (%)	− (%)	+ (%)	− (%)	+ (%)	− (%)
No biofilm	9 (18.8)	1 (2.1)	0	10 (20.8)	8 (16.7)	2 (4.2)	9 (18.8)	1 (2.1)	6 (12.5)	4 (8.3)	1 (2.1)	9 (18.8)	5 (10.4)	5 (10.4)
Weak	17 (35.4)	6 (12.5)	0	23 (47.9)	20 (41.7)	3 (6.3)	20 (41.7)	3 (6.3)	9 (18.8)	14 (29.2)	8 (16.7)	15 (31.3)	10 (20.8)	13 (27.1)
Moderate	2 (4.2)	1 (2.1)	0	3 (6.3)	3 (6.3)	0	3 (6.3)	0	3 (6.3)	0	2 (4.2)	1 (2.1)	3 (6.3)	0
Strong	10 (20.8)	2 (4.2)	0	12 (25)	10 (20.8)	2 (4.2)	12 (25)	0	9 (18.8)	3 (6.3)	5 (10.4)	7 (14.6)	8 (16.7)	4 (8.3)
Total	38 (79.2)	10 (20.8)	0	48 (100)	41 (85.4)	7 (14.6)	44 (91.7)	4 (8.3)	27 (56.3)	21 (43.8)	16 (33.3)	32 (66.7)	26 (54.2)	22 (45.8)

## Data Availability

Data are contained within the article and Supplementary Materials.

## Supplementary Materials

The following supporting information can be downloaded at: https://www.mdpi.com/article/10.3390/vetsci11040180/s1. Table S1. Antibiotic resistance patterns; Table S2. Result of biofilm assay, biofilm-associated gene screening, and AST for 48 isolates of *E. faecalis*; Table S3. Result of biofilm assay, biofilm-associated gene screening, and AST for 48 isolates of *E. faecium.*
