# Supplementary material for: Comparative Analysis of Antibiotic Resistance and Biofilm Characteristics of Two Major Enterococcus Species from Poultry Slaughterhouses in South Korea
Source: Vet Sci. 2024 Apr 16;11(4):180. doi: 10.3390/vetsci11040180 (PMC11054628; doi:10.3390/vetsci11040180)
Supplement: Supplementary file 1 [file vetsci-11-00180-s001.zip › vetsci-2854915-supplementary.pdf]

Supplementary Table S1. Antibiotic resistance patterns

Antibiotic resistance patterns	No. of *E. faecalis* (%)	No. of *E. faecium* (%)
Pan-susceptible	0 (0)	1 (2.08)
CIP	0 (0)	1 (2.08)
ERY	0 (0)	1 (2.08)
LZD	0 (0)	1 (2.08)
Q/D	5 (10.42)	1 (2.08)
ERY, Q/D	4 (8.33)	0 (0)
ERY, RIF	0 (0)	4 (8.33)
MIN, PEN	0 (0)	1 (2.08)
Q/D, RIF	6 (12.5)	0 (0)
AMP, CIP, PEN	0 (0)	2 (4.17)
AMP, PEN, RIF	0 (0)	1 (2.08)
CIP, DOX, Q/D	1 (2.08)	0 (0)
CIP, ERY, LZD	0 (0)	1 (2.08)
CIP, ERY, Q/D	1 (2.08)	0 (0)
CIP, Q/D, RIF	0 (0)	2 (4.17)
DOX, ERY, LZD	1 (2.08)	0 (0)
DOX, ERY, Q/D	1 (2.08)	0 (0)
DOX, LZD, Q/D	1 (2.08)	0 (0)
ERY, LZD, PEN	0 (0)	1 (2.08)
ERY, MIN, Q/D	4 (8.33)	0 (0)
ERY, PEN, Q/D	1 (2.08)	0 (0)
ERY, Q/D, RIF	4 (8.33)	2 (4.17)
LZD, MIN, Q/D	0 (0)	1 (2.08)
CIP, ERY, LZD, Q/D	0 (0)	2 (4.17)
CIP, ERY, LZD, RIF	0 (0)	1 (2.08)
CIP, ERY, MIN, Q/D	9 (18.75)	0 (0)
CIP, MIN, Q/D, RIF	1 (2.08)	0 (0)
DOX, ERY, MIN, PEN	0 (0)	1 (2.08)
DOX, ERY, MIN, Q/D	0 (0)	4 (8.33)
DOX, ERY, Q/D, RIF	1 (2.08)	0 (0)
DOX, LZD, MIN, Q/D	0 (0)	1 (2.08)
ERY, LZD, Q/D, RIF	0 (0)	7 (14.58)
LZD, MIN, Q/D, RIF	0 (0)	1 (2.08)
AMP, CIP, ERY, LZD, PEN	0 (0)	1 (2.08)
AMP, DOX, PEN, Q/D, RIF	1 (2.08)	0 (0)
AMP, ERY, MIN, Q/D, RIF	1 (2.08)	0 (0)
CIP, DOX, ERY, MIN, Q/D	2 (4.17)	1 (2.08)
CIP, DOX, ERY, Q/D, RIF	1 (2.08)	0 (0)
CIP, DOX, LZD, MIN, RIF	0 (0)	1 (2.08)
CIP, ERY, MIN, PEN, Q/D	1 (2.08)	0 (0)
CIP, ERY, MIN, Q/D, RIF	1 (2.08)	0 (0)
CIP, LZD, MIN, Q/D, RIF	0 (0)	1 (2.08)
DOX, ERY, LZD, MIN, Q/D	0 (0)	1 (2.08)
DOX, ERY, LZD, MIN, RIF	0 (0)	1 (2.08)
AMP, CIP, DOX, LZD, MIN, PEN	0 (0)	1 (2.08)
CIP, DOX, ERY, LZD, MIN, PEN	0 (0)	1 (2.08)
CIP, DOX, ERY, LZD, MIN, Q/D	0 (0)	1 (2.08)
CIP, DOX, LZD, MIN, Q/D, RIF	0 (0)	1 (2.08)
DOX, ERY, LZD, MIN, Q/D, RIF	0 (0)	1 (2.08)
AMP, CIP, DOX, ERY, MIN, PEN, Q/D	1 (2.08)	0 (0)

Ampicillin (AMP), ciprofloxacin (CIP), doxycycline (DOX), erythromycin (ERY), linezolid (LZD), minocycline (MIN), penicillin (PEN), quinupristin/dalfopristin (Q/D), rifampicin (RIF), and vancomycin (VAN)

Supplementary Table S2. Result of biofilm assay, biofilm-associated gene screening, and AST for 48 isolates of *E. faecalis*

Species	Biofilm		Genes							Antimicrobial Susceptibility Test (AST)											
	O. D	Quantity	*agg*	*efaA*	*bop*	*srt*	*sprE*	*cob*	*ccf*	AMP	CIP	DOX	ERY	LZD	MIN	PEN	Q/D	RIF	VAN	MDR	MAR index
E. faecalis	1.01	S	+	-	+	+	+	+	+	S	I	I	S	S	I	S	R	R	S	+	0.2
	0.95	S	+	-	+	+	+	+	+	R	S	I	R	S	R	S	R	R	S	+	0.5
	1.02	S	+	-	+	+	+	+	+	S	S	I	R	S	I	S	R	R	S	+	0.3
	1	S	+	-	+	+	+	+	+	S	I	I	R	S	I	S	R	R	S	+	0.3
	0.93	S	+	-	+	+	+	+	+	S	I	S	I	S	I	S	R	R	S	+	0.2
	0.98	S	+	-	+	+	+	+	+	S	S	I	R	S	I	S	R	I	S	+	0.2
	1.03	S	+	-	+	+	+	+	+	S	I	S	R	S	I	R	R	I	S	+	0.3
	1.12	S	+	-	+	-	+	+	+	S	I	I	R	S	I	S	R	S	S	+	0.2
	0.5	M	+	-	+	+	+	+	+	S	I	I	R	S	I	S	R	S	S	+	0.2
	1.21	S	+	-	+	+	+	+	+	S	R	R	R	S	R	S	R	S	S	+	0.5
	1.14	S	+	-	+	+	+	+	+	S	R	I	R	S	I	S	R	S	S	+	0.3
	0.69	M	+	-	+	+	+	+	+	S	I	R	R	S	I	S	R	R	S	+	0.4
	0.62	M	+	-	+	+	+	+	+	S	R	R	S	S	I	S	R	S	S	-	0.3
	1.19	S	+	-	+	-	+	+	+	R	I	R	S	S	I	R	R	R	S	+	0.5
	0.79	S	+	-	+	+	+	+	+	S	I	I	S	S	S	S	R	S	S	-	0.1
	0.79	S	+	-	+	+	+	+	+	S	I	I	R	S	I	S	R	R	S	+	0.3
	1.23	S	+	-	+	+	+	+	+	R	R	R	R	S	R	R	R	S	S	+	0.7
	1.09	S	+	-	+	+	+	+	+	S	R	R	R	S	I	S	R	R	S	+	0.5
	3.35	S	+	-	+	+	+	+	+	S	I	R	R	R	S	S	S	S	S	+	0.3
	0.99	S	+	-	+	+	+	+	+	S	R	R	R	S	R	S	R	I	S	+	0.5
	1.08	S	+	-	+	+	+	+	+	S	I	I	I	I	I	S	R	R	S	+	0.2
	0.69	M	-	-	+	+	+	+	+	S	I	I	S	S	I	S	R	I	S	+	0.1
	1.65	S	+	-	+	+	+	+	+	S	I	S	R	S	S	S	R	R	S	+	0.3
	1.33	S	+	-	+	+	+	+	+	S	I	I	R	S	R	S	R	I	S	+	0.3
	1.19	S	+	-	+	+	+	+	+	S	I	R	R	S	I	S	R	S	S	+	0.3
	0.92	S	-	-	+	+	+	+	+	S	I	I	S	S	S	S	R	R	S	+	0.2
	1.02	S	-	-	+	+	+	+	+	S	I	I	S	S	I	S	R	R	S	+	0.2
	1.26	S	+	-	+	+	+	+	+	S	I	S	I	S	I	S	R	S	S	+	0.1
	0.84	S	+	-	+	+	+	+	+	S	I	I	I	S	I	S	R	S	S	+	0.1
	0.48	M	-	-	+	+	+	+	+	S	R	I	R	S	R	S	R	S	S	+	0.4
	0.66	M	+	-	+	+	+	+	+	S	R	I	R	S	R	R	R	S	S	+	0.5
	0.97	S	+	-	+	+	+	+	+	S	R	I	R	S	R	S	R	I	S	+	0.4
	0.97	S	-	-	+	+	+	+	+	S	R	I	R	S	R	S	R	I	S	+	0.4
	1.02	S	-	-	+	+	+	+	+	S	R	I	R	S	R	S	R	I	S	+	0.4
	1.05	S	+	-	+	+	+	+	+	S	I	I	R	S	R	S	R	I	S	+	0.3
	1.02	S	+	-	+	+	+	+	+	S	I	I	R	S	R	S	R	I	S	+	0.3
	1.04	S	-	-	+	+	+	+	+	S	I	S	R	S	I	S	R	I	S	+	0.2
	1.12	S	+	-	+	+	+	+	+	S	I	I	I	S	I	S	R	I	S	+	0.1
	0.73	S	+	-	+	+	+	+	+	S	I	R	I	R	I	S	R	I	S	+	0.3
	1.18	S	-	-	+	+	+	+	+	S	I	I	I	S	I	S	R	R	S	+	0.2
	0.93	S	-	-	+	+	+	+	+	S	R	I	R	S	R	S	R	I	S	+	0.4
	1.01	S	+	-	+	+	+	+	+	S	R	I	R	S	R	S	R	I	S	+	0.4
	1.02	S	+	-	+	+	+	+	+	S	I	I	R	S	R	S	R	I	S	+	0.3
	0.69	M	+	-	+	+	+	+	+	S	R	I	R	S	R	S	R	I	S	+	0.4
	1.02	S	-	-	+	+	+	+	+	S	R	I	R	S	R	S	R	R	S	+	0.5
	1.21	S	+	-	+	+	+	+	+	S	R	I	R	S	R	S	R	I	S	+	0.4
	1.27	S	-	-	+	+	+	+	+	S	R	I	R	S	R	S	R	I	S	+	0.4
	0.63	M	+	-	+	+	+	+	+	S	R	I	I	S	R	S	R	R	S	+	0.4
Percent positive (%)			77.1	0	100	95.8	100	100	100	6.3	37.5	20.8	68.8	4.2	41.7	8.3	97.9	33.3	0	98.8	
Percent negative (%)			22.9	100	0	4.2	0	0	0	93.7	62.5	79.2	31.2	95.8	58.3	91.7	2.1	66.7	100	1.2	

Supplementary Table S3. Result of biofilm assay, biofilm-associated gene screening, and AST for 48 isolates of *E. faecium*

Species	Biofilm		Genes							Antimicrobial Susceptibility Test (AST)											
	O. D	Quantity	*agg*	*efaA*	*bop*	*srt*	*sprE*	*cob*	*ccf*	AMP	CIP	DOX	ERY	LZD	MIN	PEN	Q/D	RIF	VAN	MDR	MAR index
E. faecium	0.13	W	-	-	+	+	-	-	-	S	S	S	R	I	S	S	R	R	S	+	0.3
	0.13	W	+	-	+	+	-	-	-	S	S	S	R	I	S	S	I	R	S	+	0.2
	0.13	W	+	-	+	+	-	-	-	S	S	S	R	I	S	S	I	R	S	+	0.2
	0.13	W	-	-	+	+	-	+	-	S	S	S	R	I	S	S	I	R	S	+	0.2
	0.13	W	+	-	+	+	-	-	+	S	S	S	R	S	S	S	S	R	S	-	0.2
	0.13	W	+	-	+	+	-	+	-	S	R	R	R	S	R	S	R	S	S	+	0.5
	0.13	W	+	-	+	+	-	-	+	S	I	R	R	S	R	S	R	I	I	+	0.4
	0.14	W	+	-	+	-	-	+	+	S	I	R	R	S	R	S	R	I	I	+	0.4
	0.93	S	+	-	-	+	-	-	+	S	I	R	R	R	R	S	R	R	I	+	0.6
	0.15	W	-	-	-	+	-	-	+	S	I	R	R	I	R	S	R	I	I	+	0.4
	0.14	W	+	-	+	+	-	+	+	R	R	S	I	S	S	R	I	S	S	+	0.3
	0.15	W	+	-	+	+	-	-	-	S	R	S	I	I	S	S	R	R	I	+	0.3
	0.12	W	+	-	+	+	+	+	-	R	R	S	R	R	S	R	S	S	S	+	0.5
	0.96	S	+	-	+	+	+	+	+	S	I	S	R	I	S	S	I	S	S	+	0.1
	0.14	W	+	-	+	+	+	-	+	S	I	S	I	R	R	S	R	R	S	+	0.4
	0.57	S	+	-	+	+	+	+	+	S	I	S	S	S	S	S	I	S	S	-	0
	0.54	S	+	-	+	+	+	-	+	S	I	S	I	R	R	S	R	I	I	+	0.3
	1.22	S	+	-	-	+	+	+	+	S	R	S	S	S	S	S	I	S	S	-	0.1
	0.15	W	+	-	+	+	+	+	+	R	I	S	I	I	S	R	S	R	S	+	0.3
	0.98	S	+	-	+	+	+	-	-	R	R	R	I	R	R	R	S	S	S	+	0.6
	0.78	S	+	-	+	+	+	-	-	S	I	S	I	R	S	S	I	S	S	+	0.1
	0.85	S	+	-	+	+	+	-	-	S	R	S	R	R	S	S	S	R	S	+	0.4
	0.42	M	+	-	+	+	+	+	+	S	R	R	I	R	R	S	I	R	I	+	0.5
	0.2	W	-	-	+	+	+	+	+	S	I	R	I	R	R	S	R	I	S	+	0.4
	0.97	S	+	-	+	+	+	+	+	S	R	S	R	R	S	S	I	I	S	+	0.3
	0.17	W	+	-	+	+	+	-	-	S	R	S	R	R	I	S	R	S	S	+	0.4
	0.16	W	-	-	+	+	+	-	-	S	I	R	R	R	R	S	I	R	S	+	0.5
	0.2	W	+	-	+	-	+	-	-	S	R	I	I	R	R	S	R	R	I	+	0.5
	0.15	W	-	-	+	+	+	-	+	S	R	R	I	R	R	S	R	R	I	+	0.6
	0.3	M	-	-	+	+	+	-	+	S	I	S	R	R	S	S	R	R	S	+	0.4
	0.17	N	-	-	+	+	+	-	+	S	I	S	R	R	S	S	R	R	S	+	0.4
	0.23	W	+	-	+	+	+	-	-	S	I	S	R	R	S	S	R	R	S	+	0.4
	0.17	N	+	-	+	+	+	-	+	S	I	R	R	R	R	S	R	S	S	+	0.5
	0.16	N	+	-	+	+	+	+	+	S	I	S	R	R	S	S	R	R	S	+	0.4
	0.18	N	+	-	+	+	-	-	-	S	I	S	R	R	S	S	R	R	S	+	0.4
	0.3	W	+	-	-	+	-	-	-	S	I	S	R	R	S	S	R	R	S	+	0.4
	0.31	W	+	-	-	-	-	-	-	S	I	S	R	R	S	S	R	R	S	+	0.4
	0.17	N	+	-	-	+	-	-	-	S	I	S	R	S	S	S	R	R	S	+	0.3
	0.17	N	+	-	-	+	-	-	-	S	R	R	R	R	R	S	R	S	I	+	0.6
	0.13	N	+	-	+	+	+	-	-	S	R	S	I	I	S	S	R	R	I	+	0.3
	0.16	N	+	-	+	-	-	-	+	S	I	R	R	I	R	S	R	S	S	+	0.4
	0.17	N	+	-	+	+	+	-	+	R	R	S	I	I	S	R	S	S	S	+	0.3
	0.31	W	+	-	+	+	-	+	+	S	I	I	I	I	R	R	S	S	S	+	0.2
	0.73	M	+	-	+	+	+	+	+	S	R	R	R	R	R	R	S	S	S	+	0.6
	1	S	+	-	+	+	+	+	+	S	I	R	R	I	R	R	S	S	S	+	0.4
	0.88	S	-	-	+	+	-	-	-	S	I	S	I	I	S	S	R	S	S	+	0.1
	1.04	S	-	-	+	+	-	-	+	S	I	S	R	R	S	R	I	S	S	+	0.3
	0.18	N	+	-	+	+	+	-	-	S	I	S	I	I	S	S	S	S	S	+	0
Percent positive (%)			78.1	0	92.7	93.8	78.1	66.7	77.1	10.4	33.3	31.3	62.5	52.1	39.6	18.8	54.2	47.9	0	95.8	
Percent negative (%)			21.9	100	7.3	6.3	21.9	33.3	22.9	89.6	66.7	68.7	37.5	47.9	60.4	81.2	45.8	52.1	100	4.2	
